# Hierarchical Silica Composites for Enhanced Water Adsorption at Low Humidity

**DOI:** 10.1021/acsami.4c09456

**Published:** 2024-07-17

**Authors:** Carmen Chen, Jamie L. Salinger, Molly E. Essig, Ian M. Walton, Pasquale F. Fulvio, Krista S. Walton

**Affiliations:** School of Chemical & Biomolecular Engineering, Georgia Institute of Technology, 311 Ferst Drive NW, Atlanta, Georgia 30332, United States

**Keywords:** hierarchical silicas, adsorption, water vapor, hygroscopic salt, atmospheric water harvesting

## Abstract

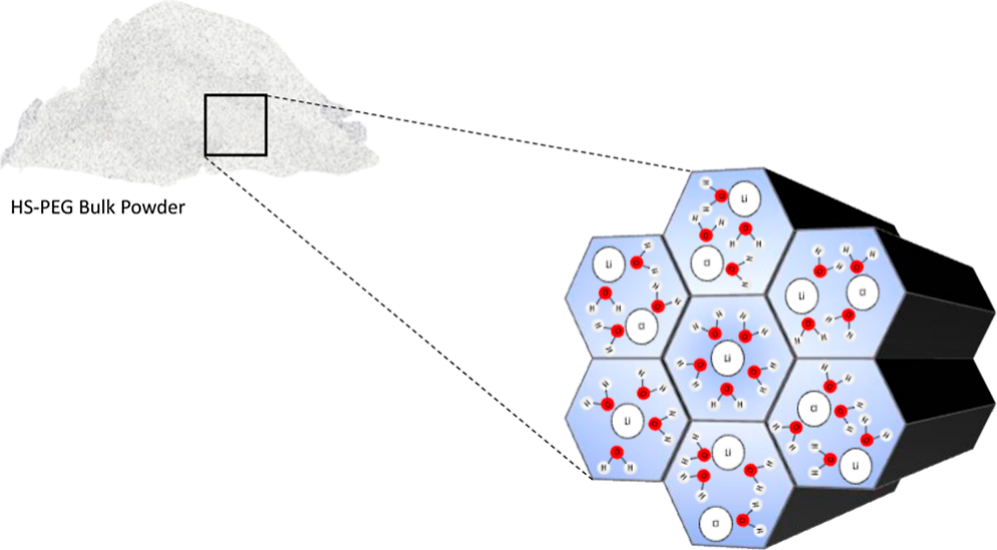

To combat water scarcity in remote areas around the world, adsorption-based atmospheric water harvesting (AWH) has been proposed as a technology that can be used alongside existing water production capabilities. However, commonly used adsorbents either have low water adsorption loadings or are difficult to regenerate. In this work, we developed two novel hierarchical silica-salt composites that both exhibit high water adsorption loadings under dry and humid conditions. The total water vapor loading, kinetics, and heats of water adsorption for both silica-salt composites were investigated. As hierarchical silicas have tunable pores and large pore volumes, these materials serve as effective host matrixes for the hygroscopic salt LiCl. Our results suggest that hierarchical pores play a significant role in water adsorption: micropores and some smaller mesopores act as “storage” sites for hygroscopic salt, whereas larger mesopores and macropores increase the accessibility of water vapor into the silica. Using this mix of pores, we achieved greater than 0.4 g H_2_O/g composite at 10% RH and 27 °C. Additionally, we found that the salt-impregnated silica and bare silica had the same heat of adsorption: 80–90 kJ/mol. The results suggest that the H-bond interactions are similar for both systems and that the primary mechanism at play here is water cluster adsorption/desorption. Despite the similar energies, the LiCl-containing materials exhibited considerably slower kinetics than bare silica materials. Of equal importance to the adsorption capacity and kinetics of these composites is their mechanical stability. To assess their mechanical stability, high-energy ball milling of silica was conducted to create more uniform particle sizes. However, reduced particle sizes came at a cost—the BET surface areas and pore volumes were drastically decreased after more than 1 h of ball milling. Findings from this study suggest that short-term ball milling may be a viable large-scale option to reduce particle size in silica materials without sacrificing significant performance.

## Introduction

An increasing number of people around the world face water scarcity, as access to surface and underground freshwater vary seasonally and geographically.^1,2^ With the number of remaining options dwindling for accessing freshwater, more nations have turned toward desalination as a source for potable water. In some remote arid regions where there is no easy access to saltwater sources, desalination can be difficult to implement and can cause major negative natural impacts.^3^ As such, there is a need to develop an additional technology that can add to the existing capabilities for water production in remote areas around the world: atmospheric water harvesting (AWH).

There is a significant amount of water vapor in the atmosphere that can be harvested to produce potable water, even with concentration gradients varying throughout the globe and with altitude.^4,5^ To capture this water, researchers have developed various AWH techniques. AWH techniques such as fog harvesting and dewing require high humidity conditions to produce potable water.^6^ Thus, they are not suitable techniques for arid regions around the world that are home to more than a third of the world’s population. A third AWH technique that can be applied to both arid and humid climates is adsorption-based AWH, where an adsorbent material is used to adsorb and desorb water vapor.

Unfortunately, some commonly used sorbents such as zeolites and silicas are either difficult to regenerate or have low water adsorption loadings, specifically at low relative humidity conditions. For example, zeolites 4A, 5A, 10X, and 13X all require regeneration temperatures from 250 to 300 °C and all have maximum water capacities of ≤0.3 g/g.^7,8^ While there are a number of metal–organic frameworks (MOFs) that can be used as atmospheric water harvesters, there still does not exist a material that can adsorb large quantities of water at both low and high humidity conditions. For example, MOFs such as UiO-66, MOF-303, and MOF-801 have maximum water capacities of ≤0.5 g/g, but in the low relative humidity regime (≤25% RH), they can only adsorb ≤0.3 g/g.^9−11^ Typically, sorbents with significant water adsorption at low relative humidity have small pore sizes and volumes that equate to a relatively low equilibrium capacity, such as with MOF-801 and MOF-303.^9^ Sorbents that have large pore sizes and volumes will adsorb high amounts of water at high humidity but exhibit low water adsorption loadings at the low relative humidity (RH) range. This phenomenon is seen with MOFs Cr-soc-MOF-1 and MIL-101(Cr). Due to their large pore volumes, these MOFs can adsorb ≥1.2 g/g but only at relative humidities above 50% RH. Below 50% RH, they are able to adsorb only ≤0.2 g/g, rendering them virtually unusable in drier environments.^9,12^ Some of the most water stressed regions experience extremely arid conditions (≤10% RH), but currently, there does not exist a sorbent that is able to adsorb large quantities of water at both low humidity and high humidity. To bridge this material development gap, we propose a novel hierarchical silica-salt composite material with favorable water–sorbent interactions at low % RH as well as having the pore volume necessary for adsorbing a significant amount of water at higher humidity conditions.

In this direction, soft-templated mesoporous silicates prepared using alkyl ionic surfactants, or triblock copolymers, with tailorable pore sizes and excellent water stability, appear as candidates for AWH technologies.^13−17^ A major challenge at approximately 24–25 °C, however, is that large pore silicas MCM-41 and SBA-15 exhibit low water capacities at % RH below 50% RH (∼0.14 g/g) and ∼70% RH (∼0.13 g/g), respectively.^18,19^ For these systems, the % RH (relative pressure) for water adsorption can be appropriately correlated to the Kelvin equation, and materials having pores larger than 4 nm exhibit condensation steps above 50% RH.^17,20^ Another key challenge to an efficient AWH process is developing an adsorbent material that can not only adsorb significant amounts of water vapor in dry conditions but also be suitable for a wide humidity range with enhanced water accessibility to the adsorption sites. Hierarchical silicas are mesoporous silicas that feature a bimodal or trimodal pore system of interconnected micro-, meso-, and/or macropores.^21^ The synthesis of soft-templated mesoporous silicas offers the advantage of combining different pore templating agents that can lead to different pore systems that are interconnected. Recent examples were reported for the self-assembly of silica precursors with alkylammonium and polyethylene glycol (PEG) surfactants. After calcination, alkylammonium surfactants templated small primary mesopores, and PEG yielded large secondary mesopores and macropores. Hence, a hierarchical pore network is induced when a mixture containing surfactant, polymer, and silicon alkoxide precursor undergoes concurrent gelation and phase separation processes. Changing the rates of gelation and phase separation can directly impact pore formation in the hierarchical silica. Such control over material properties is possible by modifying the ratio of the starting reagents or the synthesis temperature.^21−23^

Due to the tunable pore sizes and large pore volumes characteristic of hierarchical silicas, these materials serve as effective host matrixes for hygroscopic salts. Immobilizing hygroscopic salts in these porous substrates yield stable composites for water adsorption even at high relative humidity.^21^ For instance, hygroscopic salts, such as lithium chloride (LiCl) and calcium chloride (CaCl_2_), are known to adsorb significant amounts of water vapor but face issues like deliquescence, where the salt becomes liquid upon adsorbing water, and agglomeration.^12,24^ Incorporation of hygroscopic salt into porous materials has been proposed as a potential strategy to merge the benefits of both components, as the high surface area of the substrate ensures water vapor accessibility to the immobilized salts. For example, Cortés et al. successfully impregnated silica gel with LiBr, MgCl_2_, and CaCl_2_ and tested their water adsorption capacities.^25^ The authors observed an increase in the water adsorption loading at 25 °C and 39% RH for silica gel composite impregnated with 17 wt % CaCl_2_ from 0.06 to 0.33 g/g after salt impregnation. Separately, Zheng and co-workers also synthesized silica gel composites using LiCl, LiBr, and CaCl_2_.^26^ The salt impregnation improved adsorption loadings from ∼0.13 to ∼0.43 g/g at 60% RH at 20 °C for the best performing sample. In both studies, the water capacities of the silica gel composites were ultimately limited by their total pore volumes, none of which exceeded 1.26 cm^3^/g. On the other hand, composite systems having hierarchical pore structure, such as that of activated carbon fiber (ACF)-colloidal silica-LiCl composites, exhibited total water adsorption loading of up to 2.29 g/g while having a total pore volume of less than 0.07 cm^3^/g.^27^ Such differences with previous studies using silica gels potentially arise from the presence of micropores and macropores from the ACF for anchoring silica and LiCl and for water vapor diffusion, respectively. The combined effect of these pores ensure accessibility to the dispersed LiCl within the secondary mesopores of the agglomerated colloidal silica particles. Finally, the colloidal silica conferred mechanical stability to the ACF composites, as LiCl@ACF systems were found to lack mechanical rigidity in the presence of water vapor. While water adsorption in materials having unimodal or bimodal pore systems with micropores and mesopores is known, that of systems containing additional macropores has been limited to nanocomposites. Having information on the water adsorption of silicas, especially those having tailorable mesopores and with reproducible widths and pore volumes from soft-templating, could pave the way for better sorbents for AWH use. In this work, two water-stable hierarchical silicas were prepared and characterized for water adsorption after LiCl salt impregnation. Both silicas were prepared using a modified recipe for the self-assembly of silica using cetyltrimethylammonium bromide (CTAB) surfactant and PEG 35,000 polymer.^21−23^ As opposed to previous reports, the present materials were prepared in large syntheses batches of up to 50 g. It was found that upscaling this silica synthesis leads to materials having some micropores, in addition to secondary (interparticle) mesopores and macropores, whereas premixing of CTAB with PEG prior to hydrolysis of the silica source is required to yield primary mesopores interconnected to macropores by secondary mesopores. As in previous studies, increasing the ratio of CTAB with respect to that of PEG results in increased primary mesopore volumes. It has been found that the LiCl-impregnated silicas having only textural pores lead to higher water adsorption at high relative humidity. The presence of primary mesopores templated by CTAB lead to LiCl composites having comparable water vapor adsorption at low relative humidity but consisting of lesser amounts of total LiCl. The added benefit of the latter materials is the reproducibility of results given the nature of the primary mesopores templated by CTAB. The premixing of CTAB and PEG further yielded materials having greater macropore volumes. Moreover, the effect of the solvent used to impregnate these silicas with LiCl and that of silica particle size distribution for ball-milled samples were also investigated for the total water vapor loading, kinetics, and heats of water adsorption. While total loading does not correlate with the LiCl loading that is impacted by the solvent used, ball-milled silica samples had comparable kinetics due to the overall large mean particle size. Finally, all silica and LiCl-containing materials exhibit the same heats of water adsorption from microcalorimetry studies, of 80–90 kJ/mol. The results suggest that the H-bond interactions are similar for all systems and that the primary mechanism is water cluster adsorption/desorption. Despite the similar energies, the LiCl-containing materials exhibited considerably slower kinetics than bare silica materials.

The novel hierarchical silica-salt composite material presented in this work can adsorb significantly larger quantities of water at both low and high humidity conditions, able to be fully regenerated, and is stable over time. These features make this composite a promising material for implementation in AWH systems in many water scarce regions.

## Results and Discussion

### Synthesis and Characterization of Hierarchical Silicas

**Figure 1:**
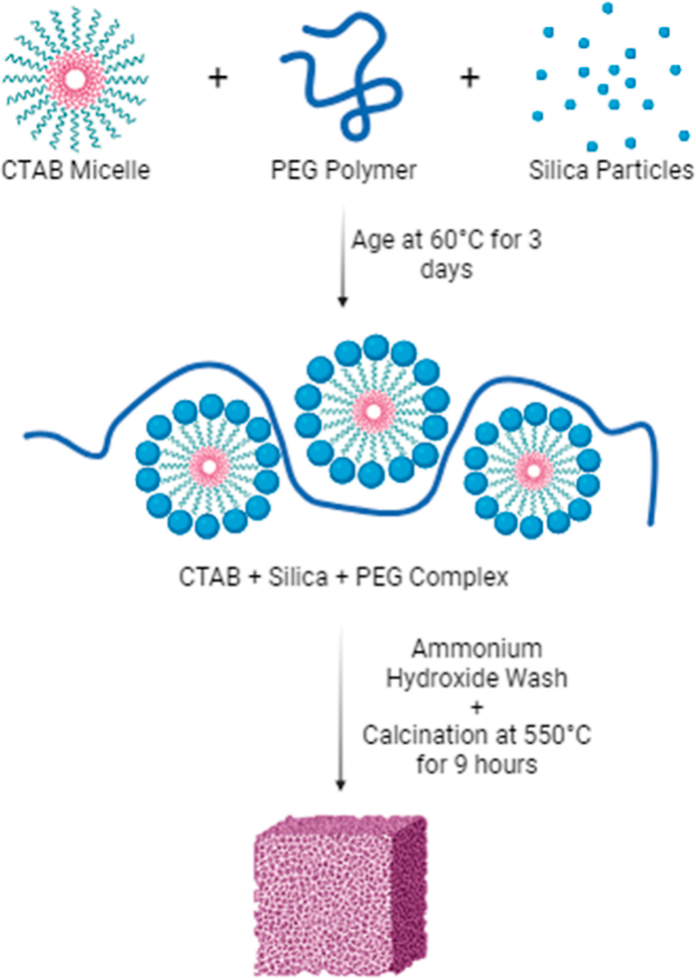
Schematic diagram of the hierarchical silica sorbent synthesis and preparation.

**Figure 2:**
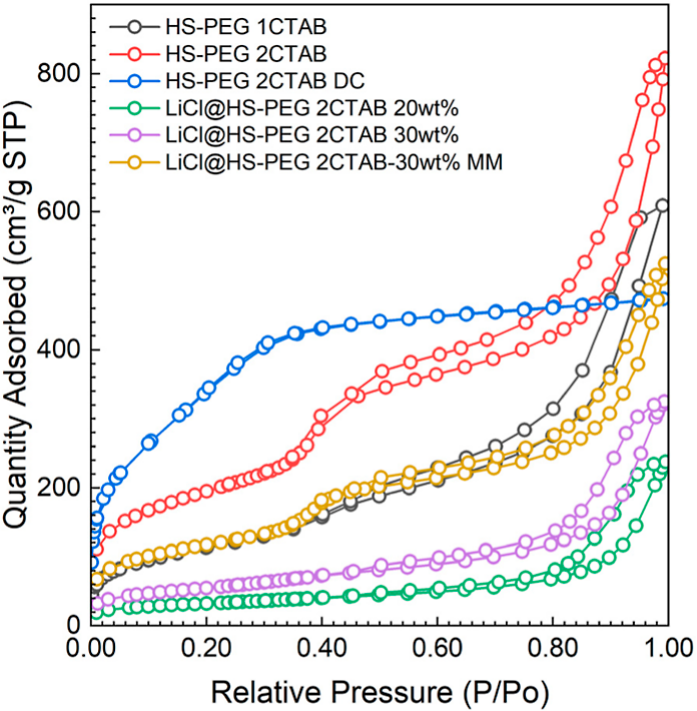
Nitrogen adsorption isotherms for HS-PEG and LiCl-impregnated HS-PEG and HS-PEG synthesized with 2× the amount of C_16_TAB (HS-PEG 2CTAB) and LiCl-impregnated HS-PEG 2CTAB. HS-PEG 2CTAB DC was directly calcined with no ammonia wash. LiCl@HS-PEG 2CTAB-30 wt % MM is a mechanical mixture of LiCl and silica (not impregnated). After impregnation with salt, the pore volume decreased in both hierarchical silicas.

**Figure 3:**
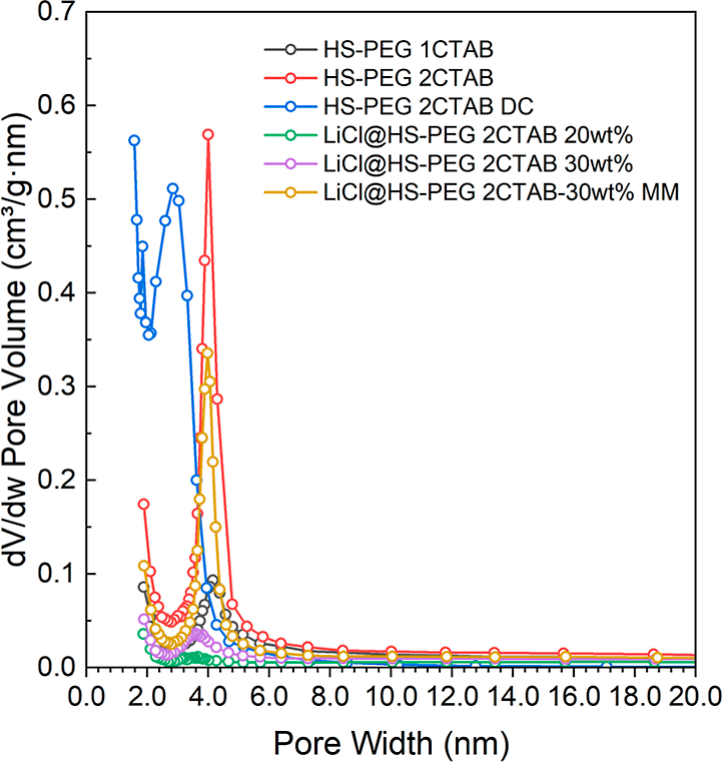
Pore size distributions are calculated using the BJH and KJS method for bare silica samples and the salt-impregnated samples from Figure 2.

Two hierarchical silicas, HS-PEG and HS-PEG 2CTAB, were synthesized using different CTAB reagent ratios and slightly different procedures as detailed in the Experimental Section and shown in Figure 1. In this synthesis, the silica sol forms around the CTAB micelles. During the first stage of drying, PEG phase segregates via spinodal decomposition.^28^ In the consecutive step of hydrothermal synthesis using aqueous NH_4_OH, the CTAB mesophase develops and the cylindrical micelles expand within the silica framework. In agreement with previous reports, the isotherm in Figure 2 for a material obtained by direct calcination in air of the composite from step 1, HS-PEG 2CTAB DC, indicates the presence of small mesopores. The isotherm is type IV, with a knee between *P*/*P*_0_ ∼ 0.2 and 0.3. The corresponding PSD in Figure 3 indicates the distribution of pores centered around 3 nm, being broader than those for the material obtained from the same CTAB ratio and the hydrothermal synthesis step. In the HS-PEG 2CTAB synthesis, the increase in CTAB reagent combined with the predissolution of CTAB with PEG prior to the addition of the silica precursor TEOS leads to a higher total mesopore volume compared to HS-PEG. To characterize the surface area and pore volume of the materials, corresponding nitrogen adsorption isotherms for the two silicas are shown in Figure 2.

Both nitrogen isotherms for HS-PEG and HS-PEG 2CTAB are type IV, characteristic of mesoporous materials. Unlike HS-PEG, two distinct capillary condensation steps are seen for HS-PEG 2CTAB at a *P*/*P*_0_ of ∼0.35 and ∼0.80, which correlate with mesopore formation by two distinct mechanisms. Specifically, the step at *P*/*P*_0_ ∼ 0.35 corresponds to mesopores templated by CTAB, whereas the *P*/*P*_0_ of 0.80 step corresponds to larger secondary mesopores templated by PEG. The hysteresis loops more closely resemble the H-2 type. These are characteristic of materials having constricted mesopores. The additional step in the desorption hysteresis for HS-PEG 2CTAB are often reported for constricted secondary slit-like pores.^29^

The corresponding pore size distribution (PSD) curves corroborate with this analysis, as the first condensation step corresponds to mesopores of approximately 4 nm in size. The second condensation step corresponds to pores in the range of 10–50 nm. The PEG polymer is polydisperse, causing the partial interpenetration of PEG chains around the silica-CTAB domains during synthesis, thus leading to a secondary soft-templating reaction.^21−23^ For the HS-PEG sample, the CTAB template did not yield similar well-defined small mesopores. Instead, the calculated PSD curve indicates only a broad distribution of large mesopores with additional micropores. The hydrolysis and segregation of the silica-PEG domains must have occurred faster than the self-assembly between silica-CTAB system, thus leading to only interparticle silica pores templated by PEG. The CTAB did not form a mesophase, and instead, it was trapped within the forming silica framework, thus yielding micropores after its removal.

The mesopore volume taken from the nitrogen adsorption isotherms at a *P*/*P*_0_ of 0.99 was 1.44 cm^3^/g for HS-PEG 2CTAB and 0.61 cm^3^/g for HS-PEG. The Brunaeur–Emmett–Teller (BET) surface areas calculated at a *P*/*P*_0_ range of 0.05–0.2 were similar for both materials as HS-PEG 2CTAB had a BET surface area of 727 m^2^/g compared to 719 m^2^/g for HS-PEG.

The total pore volume taken from mercury intrusion at 60,000 psia (Figure S1) was 4.37 cm^3^/g for HS-PEG 2CTAB and 3.38 cm^3^/g for HS-PEG. This resulted in a total macropore volume of 2.93 cm^3^/g for HS-PEG 2CTAB and 2.77 cm^3^/g for HS-PEG. The bulk density and porosity were 0.18 g/mL and 81.4%, respectively, for HS-PEG 2CTAB and 0.24 g/mL and 80.5%, respectively, for HS-PEG. These similar bulk compositions for both silicas suggest that the modified synthesis primarily serves to tune the mesopore volume of the resulting silica structure while maintaining very similar overall compositions.

### Salt Impregnation of Hierarchical Silicas

After characterization, hierarchical silicas were impregnated with 20, 25, and 30 wt % LiCl solutions to determine the best performing composite materials. LiCl was selected as it has the highest water capacity in arid conditions compared to some other hygroscopic salts.^12^ In this work, all composites are referred to with the naming convention of salt@host-matrix. The nitrogen adsorption isotherms for the optimum LiCl@HS-PEG and LiCl@HS-PEG 2CTAB samples are shown in Figure 2. Figure 2 also includes the nitrogen isotherm for a directly calcined silica sample that did not undergo the ammonia treatment as well as a sample that is a mechanical mixture of LiCl and the HS-PEG 2CTAB.

For HS-PEG, the optimum salt solution was 25 wt % LiCl salt in methanol. On the other hand, the optimum salt solutions for HS-PEG 2CTAB were 20 wt % in a 50/50 mix of water and methanol and 30 wt % in methanol. The nitrogen adsorption isotherms in Figure 2 are type IV, with the H-2 hysteresis loops. The hysteresis loop for the composite LiCl@HS-PEG is better defined than that of the HS-PEG material. As for LiCl@HS-PEG 2CTAB, the condensation step nearly disappeared, and the second step indicates a decreased pore volume. The drop in total pore volume and the loss in surface area of the silicas after LiCl impregnation suggest a pore filling mechanism with LiCl, validating the presence of salt within the porous particles. The surface areas for both 20 and 30 wt % LiCl@HS-PEG 2CTAB was 243 m^2^/g, and the surface area for LiCl@HS-PEG was 222 m^2^/g. On the other hand, the mesopore volume for 20 and 30 wt % LiCl@HS-PEG 2CTAB was reduced to 0.62 cm^3^/g, and the mesopore volume for LiCl@HS-PEG was lowered to 0.43 cm^3^/g.

To investigate the effect of LiCl impregnation on the pores of HS-PEG and HS-PEG 2CTAB, pore size distributions were calculated using the Kruk–Jaroniec–Sayari (KJS) method, which is based on the Barret–Joyner–Halenda (BJH) algorithm and calibrated for materials having small and cylindrical mesopores.^30^ The results are as shown in Figure 3.

The pore size distribution for HS-PEG is broader than that of HS-PEG 2CTAB, which follows the broad capillary condensation step seen in Figure 2, indicating a wider range of pore sizes created. After LiCl impregnation, there is a noticeable decrease in the micropores and small mesopores for both salt-impregnated silica materials. This shift in the pores suggests that LiCl is primarily contained within the smaller pores, which act as salt “storage” sites. The larger mesopores have some salt impregnated in them but to a lesser extent than that of the smaller pores. The larger mesopores are expected to increase the accessibility for both salt and water into the internal particle pores templated by CTAB.

Figures 2 and 3 also show the nitrogen isotherm and pore size distribution for a directly calcined sample of HS-PEG 2CTAB. This sample did not undergo an ammonia wash. The type I nitrogen isotherm observed shows that the mesopores are formed due to the ammonia wash removing the CTAB. The removal of CTAB during the ammonia wash results in the mesopores, which are not seen in the directly calcined sample. The pore size distribution of the directly calcined sample also shows more micropores and less mesopores, indicating the role of the ammonia wash in mesopore formation.

To further verify the presence of the LiCl in the pores of the silica material, a mechanical mixture of LiCl and silica, LiCl@HS-PEG-30 MM, was prepared and analyzed. The 30 wt % loading of LiCl in this composite was selected since it reflects an average optimum found for the present silica materials. Figures 2 and 3 show the nitrogen isotherm and pore size distribution for the mechanically mixed sample. This mechanical mixture represents a physical mixture of bulk LiCl and silica, where the LiCl is not impregnated into the primary mesopores. In the nitrogen isotherm, the decrease in the uptake reflects the amount of nonporous bulk LiCl salt in the mixture. Moreover, the capillary condensation step at a *P*/*P*_0_ of 0.35 is still present, thus verifying that the mesopores of the silica are left unfilled. In the solution-impregnated samples, this condensation step is lost due to pore filling and pore blockage by the salt particles inside the mesopores. This indicates that, in the solution-impregnated samples, most of the salt is confined within the primary pores of the silica and resides less on the external surfaces.

While water has primarily been used in other salt-impregnation studies, in this work, methanol was also selected for LiCl impregnation studies due to the hydrophobicity of HS-PEG.^24,31^ It has been previously reported that calcining mesoporous silica can increase its hydrophobicity. In calcined materials, the surface silanols condensed to form siloxane bridges (Si–O–Si) which are hydrophobic. The surface hydroxyl groups are regenerated when silicas are exposed to water vapor.^17,32,33^ Prior to that, simply using methanol as a solvent allows for increasing the amount of LiCl that intrudes into the pores of the silica materials. This hypothesis was verified by quantifying the salt content for LiCl@HS-PEG and LiCl@HS-PEG 2CTAB samples using graphite furnace atomic absorption spectroscopy (GFAAS). The best performing LiCl@HS-PEG and LiCl@HS-PEG 2CTAB samples are tabulated in Table 1, while the rest can be found in Table S1.

**Table 1:** Calculated LiCl Content and Experimental Water Loadings at 27 °C 10% RH for Select LiCl Salt Impregnations of HS-PEG and HS-PEG 2CTAB

hierarchical silica	LiCl wt % used for impregnation	solvent	LiCl wt % calculated using GFAAS	water loading at 27 °C 10% RH using 3Flex (g/g)
HS-PEG	25	water	51.4	0.401
		methanol	38.5	0.467
		50/50 water–methanol	45.9	0.309
HS-PEG 2CTAB	20	water	38.3	0.348
		methanol	36.0	0.351
		50/50 water–methanol	26.8	0.421
	30	water	49.3	0.313
		methanol	16.6	0.436

In Tables 1 and S1, the amount of LiCl content in HS-PEG was the highest in samples that had water as the solvent. A similar trend was also observed in LiCl@HS-PEG 2CTAB samples. The lone exception to this pattern was HS-PEG impregnated with 20 wt % LiCl solution, whereby the sample that had water as the solvent had the lowest amount of salt impregnated. One explanation is that this sample perhaps had more leaching of LiCl salt during the impregnation process than the other 20 wt % samples. In general, the increased loading from a water-based LiCl solution over the methanol solutions could be explained by the higher solubility of LiCl in water compared to methanol. During the salt impregnation process, it is possible some of the methanol evaporated, causing previously dissolved LiCl to partially precipitate. This loss of solvent makes it more difficult for salt to infiltrate into the porous matrix of HS-PEG, resulting in lower impregnation amounts.

### Water Adsorption Isotherms for Silica-Salt Composites

**Figure 4:**
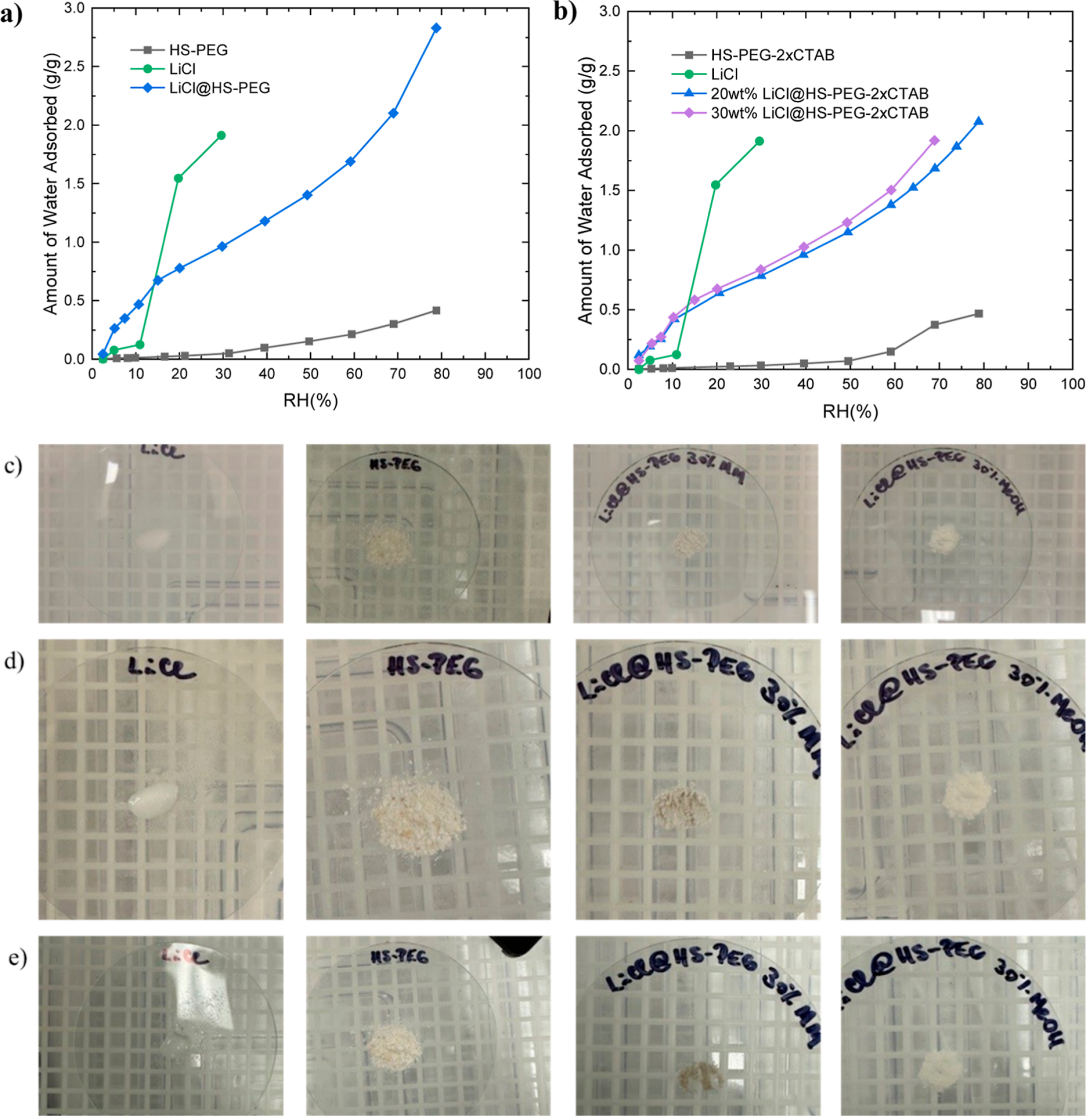
Water adsorption isotherms taken at 27 °C for (a) HS-PEG and LiCl@HS-PEG and (b) HS-PEG 2CTAB and LiCl@HS-PEG 2CTAB. In the graph on the left, a water isotherm is shown for pure LiCl. Due to the LiCl deliquescing at ∼30% RH, the instrument was unable to take additional points. Optical photographs of LiCl, HS-PEG 2CTAB, and composites prepared by mechanical mixing (MM) and by solution method using MeOH as solvent (c) before humidity exposure, (d) after 90 min of exposure to 60% RH, and (e) after increasing the humidity to 70–80% RH.

Water adsorption isotherms for best performing LiCl@HS-PEG and LiCl@HS-PEG 2CTAB samples are shown in Figure 4. Pre-exposing these samples to 50% RH water vapor is an important step as it leads to silica surface hydroxylation, with consequently increased surface hydrophilicity. This step ensures reproducibility of the adsorption by silicas and LiCl composites.^17,33^ Both silica materials exhibit the type V adsorption isotherm. This type of isotherm indicates that the interactions between adsorbate molecules are stronger than the forces between adsorbate and adsorbent surfaces.^29^ The HS-PEG isotherm has a broad condensation step that is similar to its N_2_ isotherm and can be attributed to its broad PSD. The HS-PEG 2CTAB has a well-defined step within the range of 60 to 80% RH. This range agrees with previous reports of silicas having mesopore widths of 4 to 6 nm.^15−17^

The salt-impregnated samples have a type II adsorption isotherm.^29^ Both composites greatly outperformed the unimpregnated samples across the entire humidity range of RHs measured. This enhancement in the amount adsorbed, especially at low RH, results from water loading by LiCl and is also seen in pure LiCl salt. In addition to the continuous water adsorption by the salt, multilayers of water molecules may form on the external surfaces, namely, large mesopores and macropores of the composites. Given the large mesopores found in both composites, higher RHs are required to discern the onset of the water condensation step.

Several comparative porous sorbents impregnated with LiCl are listed in Table 2 along with their water adsorption loadings under dry conditions. At low relative humidity conditions, LiCl@HS-PEG outperforms these select sorbents. Salt impregnation leads to improvements in water capacity across the whole humidity regime. The resulting water capacity of the salt@sorbent matrix not only outperforms the bare sorbent but also outperforms the bulk hygroscopic salt at high relative RHs. This indicates that there is a synergistic relationship between salt confinement and water harvesting capacity. For instance, while below 60% RH, the LiCl is found at different hydration stages, and above that humidity, the salt fully deliquesces, and forms a saturated solution. Without the support, LiCl mostly deliquesces at 47% RH.^34^ In the presence of the porous support, the salt and its solution remain confined to the pores, thus rendering a stable sorbent for cyclic use.

**Table 2:** Water Loadings of Different LiCl Supported on Microporous and to Mesoporous Sorbents in Comparison to LiCl@HS-PEG^a^

sorbent	temperature and relative humidity tested	water loading (g/g)
LiCl@HS-PEG 2CTAB	27 °C, 10% RH	0.467 g/g
LiCl/CaCl_2_@zeolite 13X^35^	20 °C, 20% RH	∼0.15 g/g
LiCl@activated carbon^36^	20 °C, 10% RH	∼0.20 g/g
LiCl@silica gel type B^26^	20 °C, 10% RH	∼0.13 g/g
LiCl@MIL-100(Fe)^37^	24.85 °C, 10% RH	∼0.13 g/g
LiCl@UiO-66(Zr)^38^	19.85 °C, 10% RH	0.271 g/g
LiCl@HKUST-1(Cu)^31^	25 °C, 30% RH	0.50 g/g
LiCl@MIL-101(Cr)^39^	25 °C, 10% RH	∼0.13 g/g

a Approximate loadings are listed for some entries using reported water isotherms.

For verifying the stability of the salt-impregnated silica composites, different samples were placed in a humidity chamber at room temperature (∼298 K) at the set relative humidity of 60%, which was then increased to 70%, and finally at 80% for different amounts of time. The optical images of the pure LiCl, HS-PEG 2CTAB, LiCl@HS-PEG 2CTAB 30 wt % MeOH, and LiCl@HS-PEG 2CTAB 30 wt % MM are provided in Figure 4c. After 90 min at 60% RH, the LiCl deliquesced and the LiCl@HS-PEG-30 MM turned to a dark gray color, and it had reduced in size from agglomeration and dissolution of free LiCl, as seen in Figure 4d. Upon increasing the relative humidity to 70–80%, LiCl and the mechanically mixed composite had fully deliquesced, while the pure silica and its solution impregnated composite counterpart did not. The latter is observed in Figure 4e. The stability of the composites prepared by solution impregnation method is attributed to most of the salt being confined within the primary mesopores of HS-PEG 2CTAB, versus the mechanically mixed sample, where all of the LiCl is on its external surfaces as bulk crystals.

Upon comparing the water vapor loadings at 27 °C 10% RH for the eight samples listed in Table 1, it appears the samples that performed the best had the lowest salt content. However, this was not necessarily the case for HS-PEG and HS-PEG 2CTAB impregnated with nonoptimum amounts of LiCl salt (Table S1). These mixed results suggest that besides the amount of salt impregnated in the material, an additional factor dictating water vapor adsorption is the potential pore-blocking by loaded LiCl. Both the amount of LiCl salt as well as the arrangement of LiCl within the pores will impact the amount of water vapor adsorbed by the material. When comparing the two silicas with each other, the salt content in the best performing LiCl@HS-PEG sample was much higher than the best performing LiCl@HS-PEG 2CTAB samples. This difference in salt content suggests that the larger mesopore volume present in HS-PEG 2CTAB plays a significant role in water vapor adsorption. The larger mesopores in HS-PEG 2CTAB allow for increased transport of water vapor into the pores compared to HS-PEG at low relative humidity. These data suggest there is a balance that must be optimized between how much salt is impregnated into the host matrix and the water vapor adsorption kinetics.

**Figure 5:**
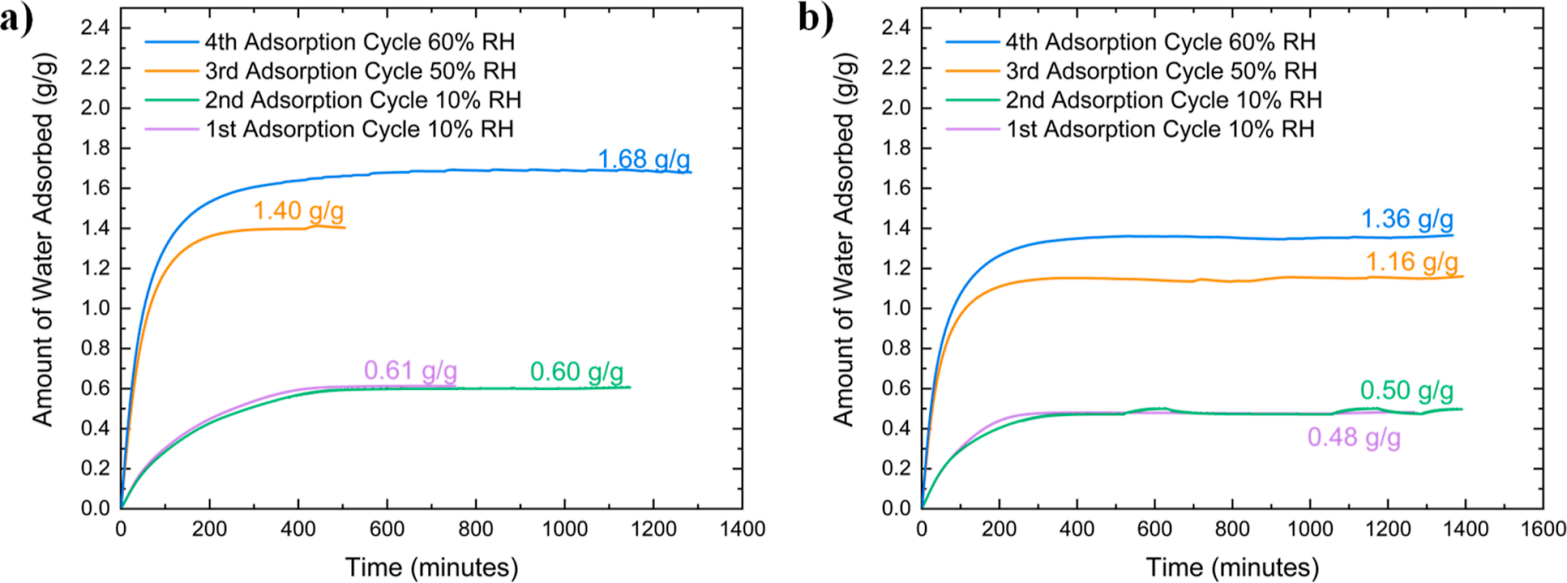
Gravimetric water adsorption cycles at 27 °C on the best performing samples (a) 25 wt % LiCl@HS-PEG impregnated in methanol and (b) 20 wt % LiCl@HS-PEG 2CTAB impregnated in 50/50 methanol–water mix. The same sample was tested throughout the four adsorption–desorption cycles. The first two adsorption–desorption cycles were at 10% RH. The third and fourth adsorption–desorption cycles were at 50% RH and 60% RH.

A necessary consideration in the development of adsorbents for AWH is their cyclic stability and subsequent regeneration. In Figure 5, the LiCl@HS-PEG and LiCl@HS-PEG 2CTAB (20 wt % in 50/50 methanol–water mix) samples that performed the best at 10% RH at 27 °C during the volumetric studies on the 3Flex were chosen for gravimetric water adsorption studies. Each sample was subjected to four consecutive adsorption–desorption cycles, with two cycles at 10% RH, one cycle at 50% RH, and the last cycle at 60% RH. Both samples were able to maintain the same water adsorption loading at 10% RH and had comparable loadings at the higher humidity range when compared to data from the 3Flex studies. At 49% RH at 27 °C, the LiCl@HS-PEG sample adsorbed 1.403 g/g and the LiCl@HS-PEG 2CTAB sample adsorbed 1.149 g/g. At 59% RH at 27 °C, the LiCl@HS-PEG sample adsorbed 1.689 g/g and the LiCl@HS-PEG 2CTAB sample adsorbed 1.378 g/g at 59% RH. These similar loadings suggest that the LiCl did not leach out of the pores of the HS-PEG during the water adsorption–desorption measurements. As mentioned before, the nanoconfinement of the salt and of the liquid solutions are instrumental for the composite sorbent performance and stability. Additionally, as the data in Figure 5 show, the LiCl@HS-PEG 2CTAB sample reached equilibrium faster than the LiCl@HS-PEG sample. This faster equilibration may be attributed to the higher mesopore volume present in the HS-PEG 2CTAB silica allowing for increased accessibility of water vapor. Notably, LiCl@HS-PEG 2CTAB had a cycle time of ∼250 min for each relative humidity tested. This is faster than the 700 min needed for LiCl@UiO-66(Zr) to reach saturation at 50% RH at 25 °C but slower than the 20 min needed for LiCl@MIL-100(Fe) at 30% RH at 25 °C.^24,37^

### Water Desorption and Heats of Adsorption Studies

**Figure 6:**
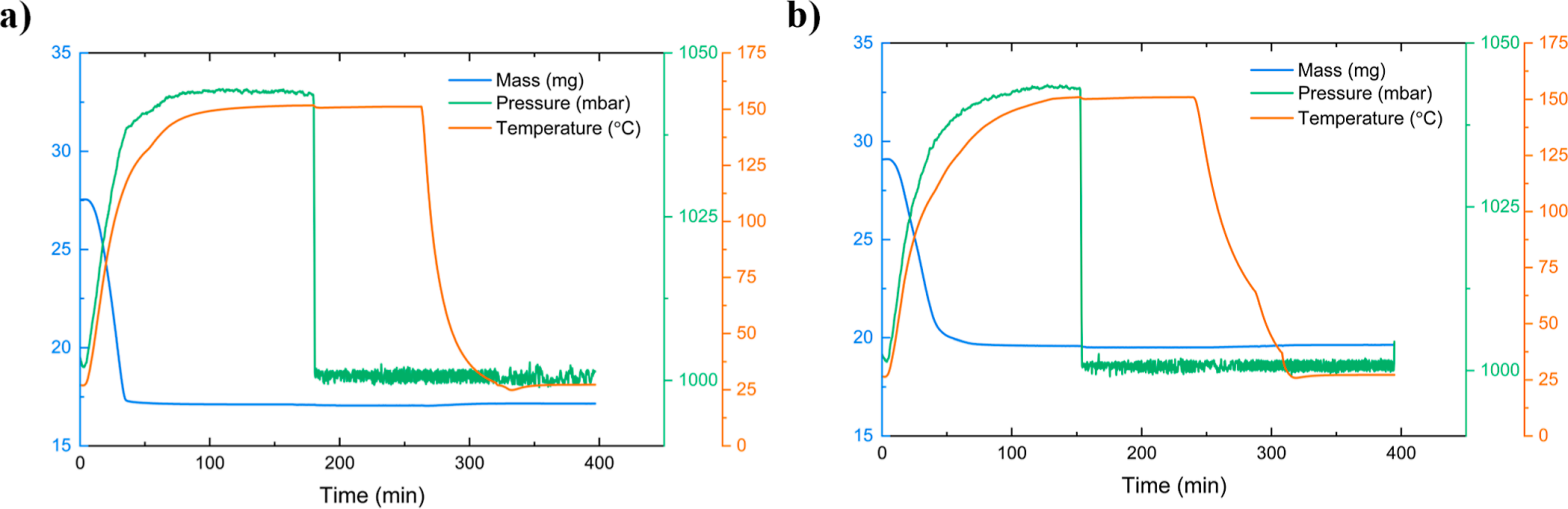
Gravimetric water desorption at 150 °C on best performing samples after adsorption at 10% RH and 27 °C (a) 25 wt % LiCl@HS-PEG impregnated in methanol and (b) 20 wt % LiCl@HS-PEG 2CTAB impregnated in 50/50 methanol–water mix. Figures show how mass of sample decreased due to increasing temperature.

In AWH, the amount of water able to be desorbed from a sorbent is equally as important as the water capacity of the sorbent. Samples used in cycling analysis, LiCl@HS-PEG (25 wt % in methanol) and LiCl@HS-PEG 2CTAB (20 wt % in 50/50 methanol–water mix), were chosen for desorption analysis. After exposure to 10% RH at 27 °C, samples were heated to 150 °C under static conditions (no purge flow and no vacuum) for temperature swing desorption. Once the sample was fully desorbed, a purge stream was introduced to decrease the pressure and flush humid air out of the system to prep the sample for another cycle of adsorption. The temperature also decreased back to 27 °C following complete desorption. In Figure 6, the desorption of LiCl@HS-PEG (25 wt % in methanol) and LiCl@HS-PEG 2CTAB (20 wt % in 50/50 methanol–water mix) is shown. As the temperature of each sample was increased to 150 °C, the mass of the sample decreased, eventually reaching the starting mass of the sample after activation and prior to adsorption, as shown in Table 3. Additionally, complete desorption occurred relatively quickly (∼90 s). This indicates that even with a high water loading at low humidity, the salt-impregnated silicas are able to be fully regenerated relatively quickly, resulting in a high-water harvesting efficiency. Similarly, since there was no significant mass change in the samples after activation and after each desorption cycle, it can be concluded that the LiCl is stable in the pores and does not leach out of the sample and that all the adsorbed water is able to be released. The heats of adsorption for the selected HS-PEG and LiCl@HS-PEG samples were discerned through isothermal adsorption studies at a constant flow rate of 200 mL/min in a TGA/DSC instrument outfitted with a humidity generator and a water vapor furnace. The quantity of water adsorbed was determined gravimetrically, while the heat flow throughout the adsorption process was monitored (Figures S10–S14). The calculated enthalpy of adsorption was found to range from 80 to 85 kJ/mol for HS-PEG and LiCl@HS-PEG samples (Table S2). The similarity in the adsorption enthalpy suggests that the host–guest interactions of the adsorption at 10% RH are similar in strength. Interestingly, the data confirm an increased time to saturation in the LiCl@HS-PEG samples, which is being attributed to the kinetics of the water-LiCl hydration process.

**Table 3:** Sample Weights after Activation and after Each Desorption Cycle^a^

hierarchical silica	cycle	mass (g)
25 wt % LiCl@HS-PEG	post activation	17.11
	post cycle 1 (10% RH)	17.12
	post cycle 2 (10% RH)	17.12
	post cycle 3 (50% RH)	17.12
	post cycle 4 (60% RH)	17.15
20 wt % LiCl@HS-PEG 2CTAB	post activation	19.56
	post cycle 1 (10% RH)	19.64
	post cycle 2 (10% RH)	19.61
	post cycle 3 (50% RH)	19.62
	post cycle 4 (60% RH)	19.58

a Activation and cycle desorption were performed at 150 °C on the best performing samples. Adsorption cycle humidity is shown in parentheses next to the cycle number. The table shows how mass of the sample returned to the starting weight, indicating complete desorption with no salt leaching or sample degradation.

### Ball-Milling HS-PEG for Uniform Particle Sizes

Prior to implementation into real-life applications, one of the key material postprocessing considerations is optimization of the particle size of the sorbent material. For example, smaller and more uniform particle sizes may be needed for more efficient packing of the adsorbent material. One method for creating smaller particle sizes is through high-energy ball milling. In the literature, ball milling has been used to break down zeolites and other materials into smaller particles.^40−43^ For example, Laranjo et al. used high-energy ball milling to reduce the particle sizes of TiO_2_/SiO_2_ xerogel powders so that they could be packed better into dye-sensitized solar cells.^44^ Ko et al. used ball milling to create a blend of the MOF M_3_HHTP_2_ and graphite prior to adding the composite material into toxic gas sensors.^45^ However, ball milling has not yet been evaluated for its potential to create uniform and structurally stable adsorbent particles for AWH applications. To this end, HS-PEG was ball-milled at various time lengths from 30 min to 12 h using a SPEX CertiPrep 8000 M Mixer/Mill.

To investigate the effect of ball milling time on the structural stability of the hierarchical silica HS-PEG, N_2_ physisorption isotherms at 77 K were measured for HS-PEG samples ball-milled at seven different time points ranging from 30 min to 12 h (Figure S2). N_2_ physisorption isotherms indicate a loss in BET surface area and pore volume of HS-PEG after just 30 min of ball milling. At 12 h of ball milling, almost no BET surface area and pore volume remain in HS-PEG, as seen in Table 4. Additionally, starting at 2 h of ball milling, the HS-PEG lost 70% of its original surface area and 76% of its original pore volume. Based on this data, it appears that the structural integrity of HS-PEG cannot be maintained past 1 h of ball milling. Hg porosimetry measurements indicate very similar bulk properties between the 30 min and 1 h samples. The 30 min ball-milled sample has a total pore volume of 1.39 mL/g, a bulk density of 0.47 g/mL, and a porosity of 65.4%. On the other hand, the 1 h ball-milled sample has a total pore volume of 1.33 mL/g, a bulk density of 0.47 g/mL, and a porosity of 62.9%. Additionally, an approximate particle size distribution was also determined from the SEM images of ball-milled HS-PEG (Figures S3–S9) taken with a Hitachi SU8230 instrument. The approximate particle radius listed in Table 4 was estimated by halving the diameter of the largest particle present in SEM images. Generally, a gradual decrease in particle size across the ball-milled samples was seen until a plateau is reached at ∼10 nm after 4 h of ball milling.

**Table 4:** BET Surface Areas and Pore Volumes for 0–12 h Ball-Milled HS-PEG^a^

ball mill (h)	BET surface area (m^2^/g)	pore volume (cm^3^/g) at *P*/*P*_0_ = 0.90	approximate particle radius (μm)
0	770	0.975	50
0.5	544	0.434	22.5
1	400	0.367	19
2	236	0.231	12.5
3	169	0.175	15
4	88	0.099	10
5	106	0.124	10
12	66	0.085	10

a Approximate particle radius determined by selecting the largest particle radius in SEM images taken of the samples.

## Conclusions

In conclusion, two novel hierarchical silicas HS-PEG and HS-PEG 2CTAB were synthesized with different amounts of mesopores and mesopore volume and impregnated with the hygroscopic salt LiCl to generate hierarchical silica-salt composite materials for tailored AWH applications. The resulting composites harness the benefits of both the salt and the sorbent alone. With the inclusion of the LiCl in the micropores and mesopores in the host silica matrix, the ensuing novel silica-salt composites showed enhanced water adsorption behavior across the entire humidity range, with no salt leaching, complete regeneration, and cyclical stability, indicating their potential for long-term AWH applications. Notably, the water loading seen at 10% RH at 27 °C (>0.4 g/g) meets or outperforms similar sorbents, specifically salt-MOF materials, tested at temperatures ranging from 20 to 30 °C.^12,37,38^ The enhanced water loadings at both low and high humidity indicate that these composites can be implemented into AWH devices for water harvesting in a wide range of environments, something previously unable to be attained, which enhances the novelty of these materials for AWH. Additionally, due to the increased number of mesopores and mesopore volume in HS-PEG 2CTAB, the composite silica-salt material exhibited faster adsorption kinetics than HS-PEG. These faster kinetics suggest that mesopores play a role in increasing the accessibility of water vapor into the composite silica-salt material. Furthermore, high-energy ball milling of HS-PEG was conducted to create more uniform particle sizes. However, reduced particle sizes came at a cost—the BET surface areas and pore volumes were drastically decreased after more than 1 h of ball milling. Interestingly, HS-PEG ball-milled for 30 min and 1 h still featured significant porosity. The findings from this study suggest that short-term ball milling may be a viable large-scale option to reduce particle size in silica materials without sacrificing significant performance. In general, the results highlight the need to evaluate the mechanical stability of adsorbents in parallel with other characterization techniques prior to their use for AWH applications. Ultimately, the findings from this study may be applied to other hierarchical materials to develop the next generation of AWH adsorbents.

## Experimental Section

### Materials

Polyethylene glycol (PEG, 35,000 g/mol), nitric acid (70%), tetraethylorthosilicate (TEOS, 98%), cetyltrimethylammonium bromide (CTAB, n-16, ≥98%), ammonium hydroxide (28–30% NH_3_ basis), and lithium chloride (≥99%) were purchased from Sigma-Aldrich.

### Synthesis of HS-PEG

The synthesis procedure is adapted from Smått et al., with some modifications.^22^ Into a beaker, 9.8068 g of polyethylene glycol (PEG, 35,000 g/mol, Sigma-Aldrich) was added. While under gentle stirring, small amounts of reagents were dosed into the beaker in the order written: 137 μL of H_2_O, 8.2 μL of HNO_3_ (Sigma-Aldrich, 70%), and 0.116 mL of tetraethoxysilane (TEOS, Sigma-Aldrich, 98%). Subsequently, 109.2 mL of H2O and 6.526 mL of HNO_3_ were poured into the beaker. Once the solution turned clear, 92.1 mL of TEOS was added. After the TEOS completely dissolved, 13.5389 g of cetyltrimethylammonium bromide (CTAB, Sigma-Aldrich, ≥98%) was added. After the solution turned clear again, the beaker with sol was placed in a programmable oven for 3 days at 40 °C. During this time, the sol slowly turned into one cylindrically shaped yellow and white solid precipitate. For the next step, the precipitate is broken up into chunks gently using a spatula. These chunks are added to a 100 mL Teflon liner until they reached 1/2 in. from the top of the liner. 20 mL of 1 M NH_4_OH (Sigma-Aldrich, 28–30% NH_3_ basis) solution is added to the Teflon liner. The Teflon liners are sealed up in autoclaves and subsequently placed in an oven for 9 h at 90 °C.

### Synthesis of HS-PEG 2CTAB

54.575 mL of H_2_O was added into a round-bottom flask suspended in a silicone oil bath. To the water, 2.299 mL of 70% HNO_3_ was added to produce an aqueous nitric acid solution. To the nitric acid solution, 2.3594 g of 35,000 g/mol PEG was added under stirring and subsequently stirred until the PEG was completely dissolved and a clear solution remained. Next, 14.0571 g of CTAB was added into the flask under stirring. The solution was then heated to 60 °C to assist in the dissolution of CTAB into the solution. Once the CTAB was completely dissolved, the mixture was lifted out of the silicone oil bath and allowed to cool back down to room temperature. Once cooled, 46.05 mL of TEOS was slowly poured into the flask under stirring. Once the mixture was clear, it was poured into a beaker and allowed to age uncovered in an oven at 40 °C for 72 h. Following the completion of the aging process, the monolith was broken up into chunks with a spatula. Next, the monolith chunks were washed with a 1 M NH_4_OH solution at 90 °C for 9 h. To a 47 mL Teflon liner, 25 mL of total liquid was added (23.26 mL H_2_O and 1.720 mL of 1 M NH_4_OH), and the monolith chunks were added into the Teflon liner until they were completely covered with 1 in. of solution above the monolith. The Teflon liners were sealed in autoclaves and placed in an oven at 90 °C for 9 h.

### Solvent Wash and Calcination of Monoliths after Ammonia Treatment

After cooling back down to room temperature, the silica monoliths were poured into a clean beaker and subsequently drained of residual solution. To the monoliths, a 0.1 M HNO_3_ solution was added. After 10 min with occasional stirring, the nitric acid solution was drained. Next, the monoliths were washed with a 25% ethanol and water solution. After soaking for 10 min, the solution was drained. This process was repeated 3 times. Next, the beaker of monolith was covered with aluminum foil (with holes for venting) and dried in an oven at 60 °C for 72 h. Following the drying process, the monoliths were calcined at 550 °C for 5 h with a ramp rate of 1 °C/min.

### LiCl Salt Impregnation of Monoliths

Into a 100 mL beaker, lithium chloride (LiCl, Sigma-Aldrich, ≥99%) was added (amounts depending on solution wt % desired) along with 50 g of solvent. The solvents were either 100% methanol, 100% water, or a 50/50 mix of methanol and water. The solution was then stirred until the LiCl was completely dissolved. Once dissolved, HS-PEG or HS-PEG 2CTAB was added into the beaker at a ratio of 5 mg of adsorbent per 1 mL of solvent. The mixture is then left to gently stir for 24 h. Following impregnation, the impregnated HS-PEG is collected from the LiCl solution and dried in an oven at 110 °C for 24 h. In this work, all composites will be referred to with the naming convention of salt@host-matrix. Next, the LiCl@HS-PEG composites are washed in a sealed humidity chamber set to 50% RH (ambient temperature) for 24 h to remove external salt from the surface of the material. Finally, the LiCl@HS-PEG is dried in an oven at 110 °C for 24 h. This wash and drying process is repeated one additional time for a total of two washes for each LiCl@HS-PEG sample. For comparison, a sample of HS-PEG 2CTAB was mechanically mixed with LiCl salt using mortar and pestle without any solvents. This sample was labeled LiCl@HS-PEG 2CTAB-30 wt % MM.

### Nitrogen Adsorption Isotherms

Nitrogen adsorption measurements at 77 K were obtained using a Micromeritics TriStar II Plus surface area and porosity analyzer (Micromeritics, Norcross GA), and a 3Flex volumetric analyzer (Micromeritics, Norcross, GA). Prior to measurements, all samples were outgassed under vacuum and at 150 °C overnight. The total pore volumes were obtained directly from the adsorption isotherms at the *p*/*p*_0_ of 0.90–0.95, and the total surface areas are calculated using the BET method within the *p*/*p*_0_ range of 0.05–0.20.^29^ The pore size distributions were calculated using the BJH KJS method.

### Mercury Porosimetry

Mercury porosimetry measurements were conducted at Micromeritics Instrument Corp. using a Micromeritics MicroActive AutoPore V 9600 version. Mercury intrusion was measured from a pressure of 0.10 to 61,000 psia to obtain pore data from 1000 to 0.001 μm at 18.63 °C. Prior to measurements, all samples were degassed at 150 °C for 5 h.

### Water Vapor Adsorption Isotherms

Water vapor adsorption isotherms at 27 °C were obtained using a Micromeritics 3Flex Surface Characterization Analyzer. Prior to water measurements, all samples were activated at 150 °C for 14 h under vacuum. For the cycling studies at 27 °C, a Hiden Isochema IGA-3 was used instead of the Micromeritics 3Flex Surface Characterization Analyzer. Samples were activated in situ at 150 °C under vacuum overnight, until no significant weight change could be detected. Air was used as the carrier gas for water adsorption measurements on the IGA-3 to best match real environmental conditions. After adsorption measurements, the samples were reactivated in situ at 1 bar and 150 °C.

### Graphite Furnace Atomic Absorption Spectroscopy

Approximately ∼5 mg of LiCl@HS-PEG or LiCl@HS-PEG 2CTAB were dissolved in 2 mL of 4 M potassium hydroxide (KOH), diluted to 25 mL. 1 mL of the solution is diluted to 10 mL, creating an effective dilution of ∼5 mg of sorbent in 250 mL of solution. Samples were analyzed in a Shimadzu 7000 series Graphite Furnace Atomic Absorption Spectrometer with a lithium lamp. The LiCl amount was determined from the Li quantification, and the LiCl quantification per mass of sorbent was derived from the total weight of the sample.

### Ball-Milling of HS-PEG

Samples of HS-PEG silica were divided into separate 2 g samples and ball-milled for varying amounts of time, between 30 min and 12 h, using a SPEX CertiPrep 8000 M Mixer/Mill. The silica was dry-loaded into a silicon nitride vial along with two 1.3 cm silicon nitride balls. The inner diameter of the silicon nitride vial is 3.8 cm, and the height of the vial is 6.7 cm. The 8000 M Mixer/Mill is a high-energy ball mill that can grind 0.2 to 10 g of sample at a time.^16^ It operates by shaking the vial back-and-forth in a three-dimensional swing that resembles a figure-8 motion.^16,17^ After ball-milling, the fine-powdered sample was allowed to cool prior to collection.

### Scanning Electron Microscopy

A Hitachi SU-8230 SEM was used to collect the images of ball-milled silica. Silica samples were placed on top of carbon tape. The Hitachi SU-8230 SEM uses a cold field emission gun, one of three possible electron guns that can be used. Cold field emission guns emit a brighter beam and need a better vacuum compared to tungsten hairpin filament guns and lanthanum hexaboride filament guns.

## Supporting Information Available

Graphite furnace atomic absorption spectroscopy data, mercury intrusion plots, nitrogen adsorption isotherms, scanning electron microscopy images, and water adsorption and enthalpy data (PDF)

## Abbreviations

ACF: activated carbon fiber
AWH: atmospheric water harvesting
CaCl_2_: calcium chloride
CTAB: cetyltrimethylammonium bromide
HS-PEG: hierarchical silica, polyethylene glycol
H_2_O: water
LiCl: lithium chloride
LiBr: lithium bromide
MeOH: methanol
NLDFT: nonlocal density functional theory
RH: relative humidity
