# Supplementary material for: Hierarchical Silica Composites for Enhanced Water Adsorption at Low Humidity
Source: ACS Appl Mater Interfaces. 2024 Jul 17;16(30):40275–85. doi: 10.1021/acsami.4c09456 (PMC11299149; doi:10.1021/acsami.4c09456)
Supplement: Supplementary file 1 — am4c09456_si_001.pdf [file am4c09456_si_001.pdf]

Supporting Information

Hierarchical silica composites for enhanced water adsorption at low humidity

Carmen Chen[1], Jamie L. Salinger[1], Molly E. Essig[1], Ian M. Walton[1], Pasquale F. Fulvio[1], Krista S. Walton[1,]*

[1]School of Chemical & Biomolecular Engineering, Georgia Institute of Technology, 311 Ferst Drive NW, Atlanta, Georgia 30332, USA

Corresponding Author

*krista.walton@chbe.gatech.edu

Table of Contents

Table S1: LiCl impregnation of HS-PEG and HS-PEG-2xCTAB characterization 3

Figure S1: Non-Local Density Functional Theory Pore Size Distributions 4

Figure S2: Mercury intrusion plot of HS-PEG and HS-PEG-2xCTAB 5

Figure S3: Nitrogen adsorption isotherms of ball milled HS-PEG 6

Figure S4: SEM of HS-PEG ball milled for 30 min 7

Figure S5: SEM of HS-PEG ball milled for 1 h 8

Figure S6: SEM of HS-PEG ball milled for 2 h 9

Figure S7: SEM of HS-PEG ball milled for 3 h 10

Figure S8: SEM of HS-PEG ball milled for 4 h 11

Figure S9: SEM of HS-PEG ball milled for 5 h 12

Figure S10: SEM of HS-PEG ball milled for 12 h 13

Adsorption enthalpy calculation procedure 14

Table S2: Adsorption enthalpies for bare/impregnated HS-PEG and HS-PEG-2xCTAB 15

Figure S11: TGA/DSC plots for HS-PEG 16

Figure S12: TGA/DSC plots for HS-PEG-2xCTAB 17

Figure S13: TGA/DSC plots for 30wt% LiCl in MeOH HS-PEG-2xCTAB 18

Figure S14: TGA/DSC plots for 20wt% LiCl in H_2O/MeOH HS-PEG-2xCTAB 19

Figure S15: TGA/DSC plots for 25wt% LiCl in MeOH HS-PEG 20

Table S1. 20wt% and 30wt% LiCl impregnations of HS-PEG and 25wt% LiCl impregnation of HS-PEG-2xCTAB with GFAAS and water uptake at 27°C and 10% RH data.

Hierarchical Silica	LiCl wt% Used for Impregnation	Solvent	LiCl wt% calculated using GFAAS	Water Uptake at 27°C 10% RH using 3Flex (g/g)
HS-PEG	20	Water	33.4	0.233
		Methanol	35.1	0.329
		50/50 Water Methanol	42.5	0.309
	30	Water	56.4	0.256
		Methanol	48.2	0.368
		50/50 Water Methanol	54.8	0.206
HS-PEG-2xCTAB	25	Water	36.9	0.337
		Methanol	24.9	0.266
		50/50 Water Methanol	30.0	0.318

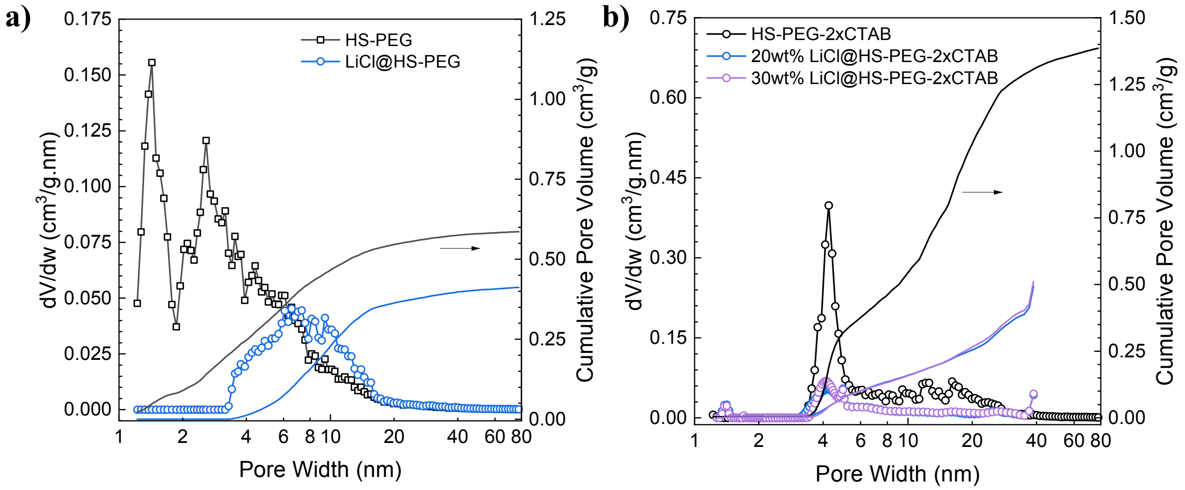

Figure S1. Pore size distributions calculated using the Non-Local Density Functional Theory (NLDFT) method for a) HS-PEG and LiCl impregnated HS-PEG and b) HS-PEG synthesized with 2x the amount of C_{16}TAB (HS-PEG-2xCTAB) and LiCl impregnated HS-PEG-2xCTAB. The solid lines are cumulative pore volumes of the materials

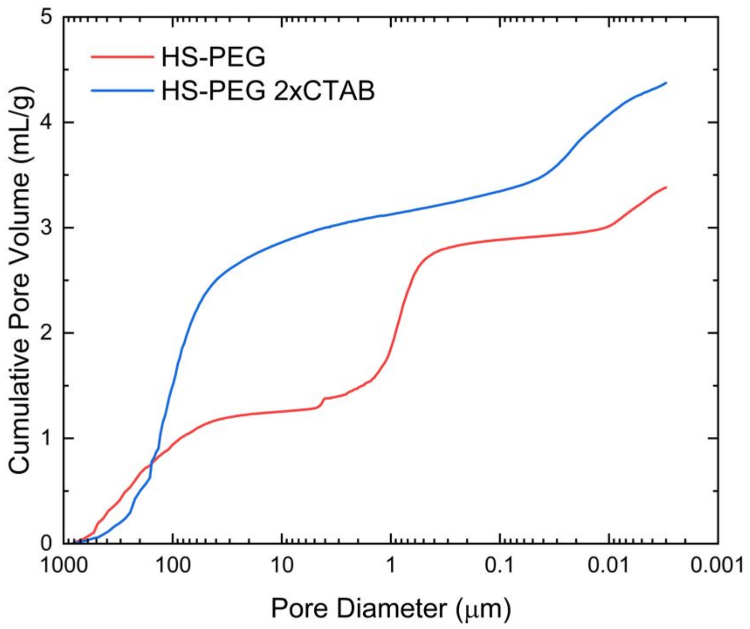

Figure S2. Mercury intrusion plots for HS-PEG and HS-PEG 2xCTAB. Mercury intrusion analysis was completed from 0.10 to 61,000 psia, with a mercury temperature of 18.63°C. Samples were heated under vacuum at 150°C for 5 hours prior to analysis. X axis is in logarithmic scale.

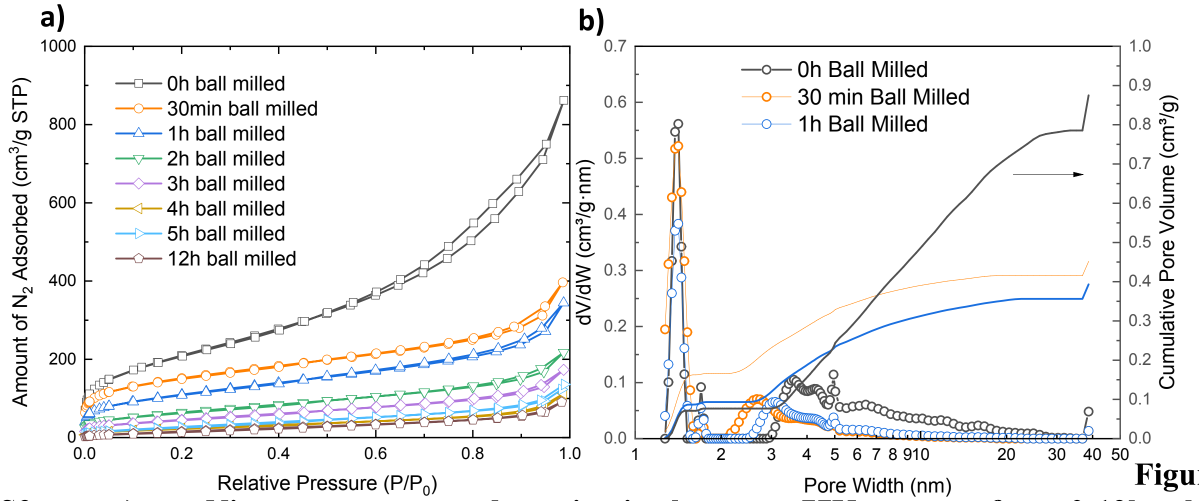

Figure S3. a) Nitrogen adsorption isotherms at 77K for 0-12h ball-milled HS-PEG, and b) Pore size distributions for 0h, 30min, 1h ball-milled HS-PEG calculated using non-local density functional theory (NLDFT). Solid lines are cumulative pore volume.

Below are the SEM images of 30 minutes to 12 hours ball-milled HS-PEG.

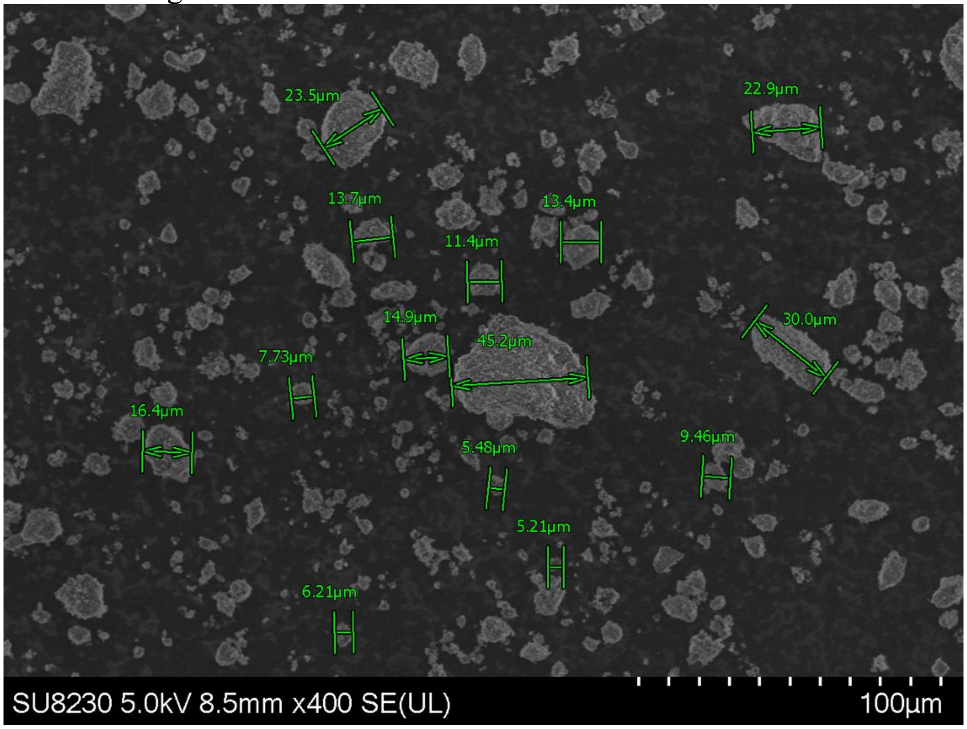

Figure S4. Measured particle sizes for 30 minutes ball-milled HS-PEG using SEM. The approximate particle radius for this sample was estimated by halving the diameter of the largest particle in the image (~45 μm).

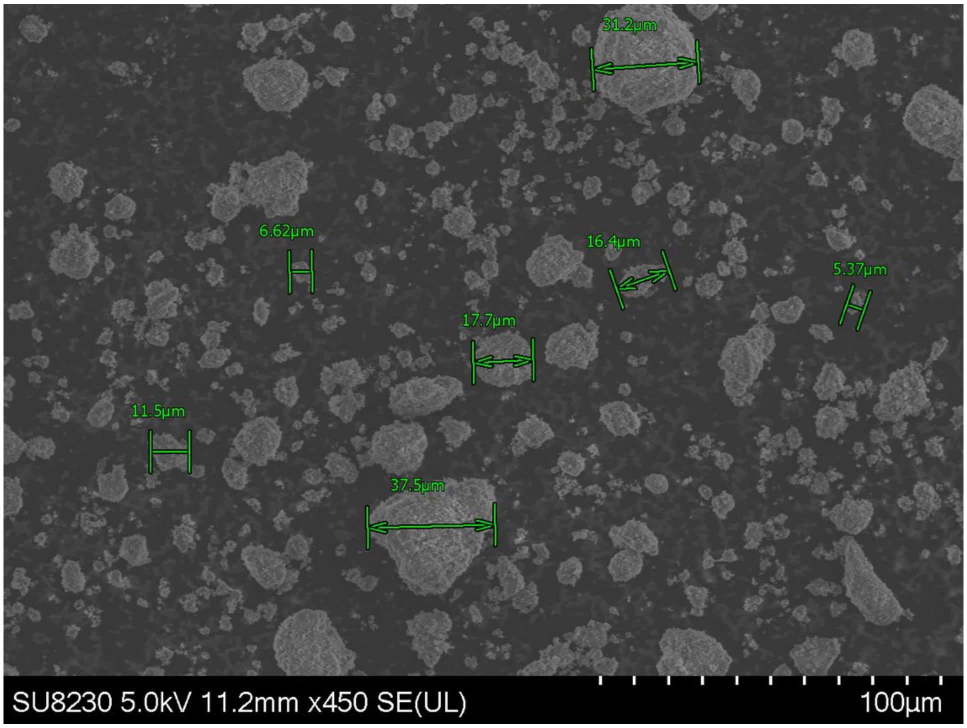

Figure S5. Measured particle sizes for 1h ball-milled HS-PEG using SEM. The approximate particle radius for this sample was estimated by halving the diameter of the largest particle in the image (~38 µm).

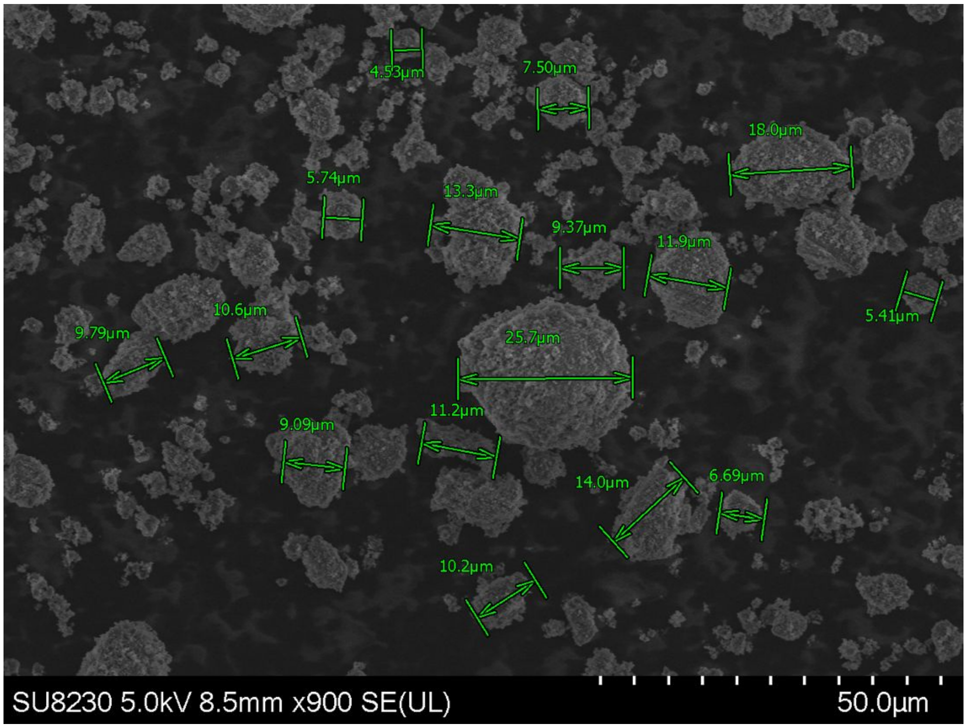

Figure S6. Measured particle sizes for 2h ball-milled HS-PEG using SEM. The approximate particle radius for this sample was estimated by halving the diameter of the largest particle in the image (~25 μm).

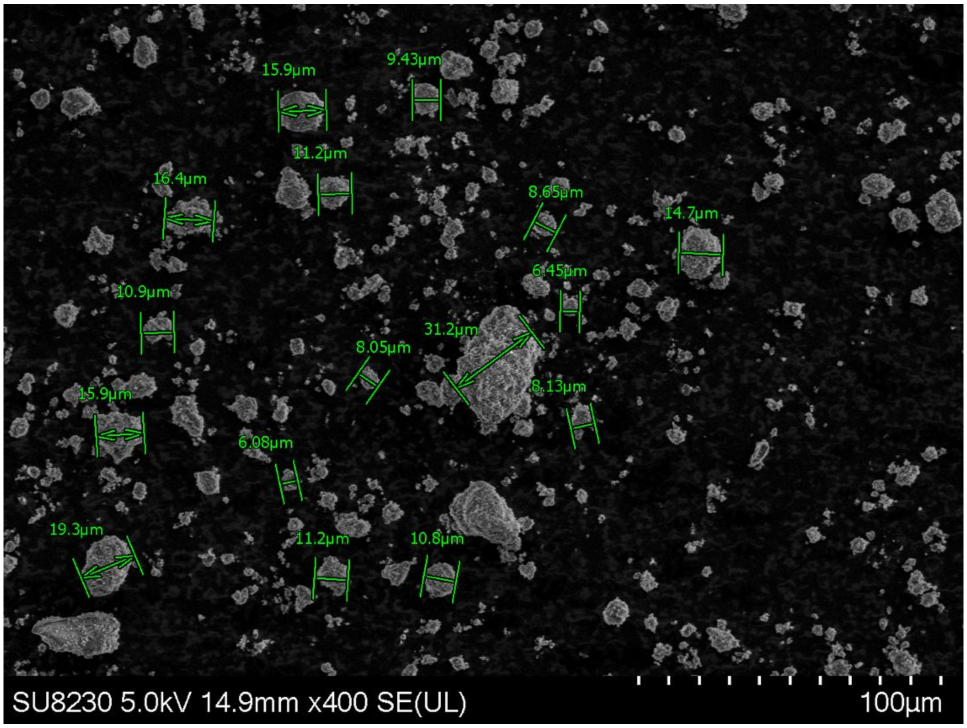

Figure S7. Measured particle sizes for 3h ball-milled HS-PEG using SEM. The approximate particle radius for this sample was estimated by halving the diameter of the largest particle in the image (~30 µm).

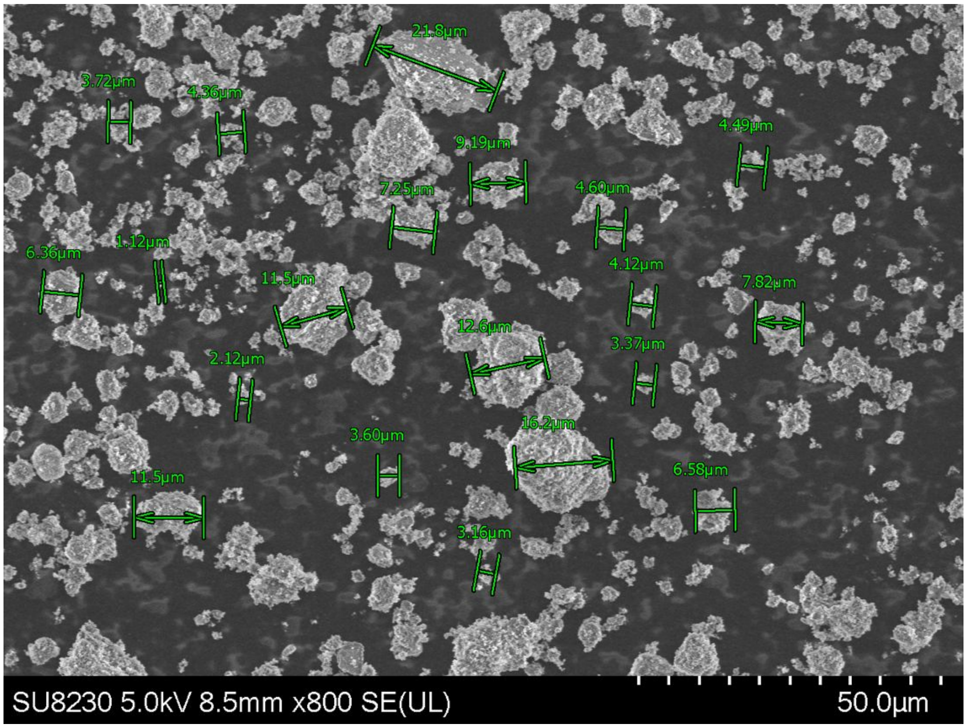

Figure S8. Measured particle sizes for 4h ball-milled HS-PEG using SEM. The approximate particle radius for this sample was estimated by halving the diameter of the largest particle in the image (~20 µm).

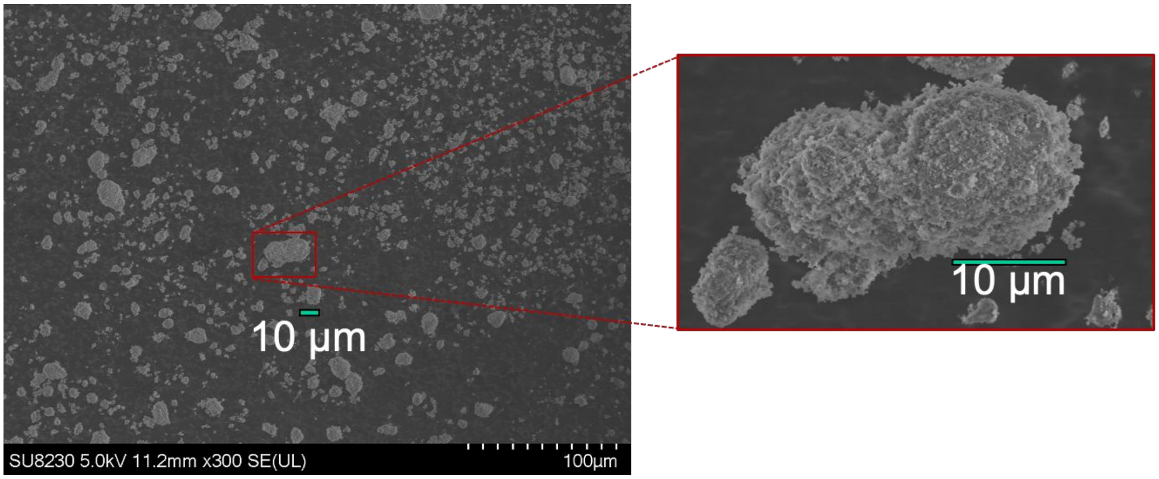

Figure S9. SEM for 5h ball-milled HS-PEG with zoomed-in view on the right. The approximate particle radius for this sample was estimated by halving the diameter of the largest particle in the image (~20 μm).

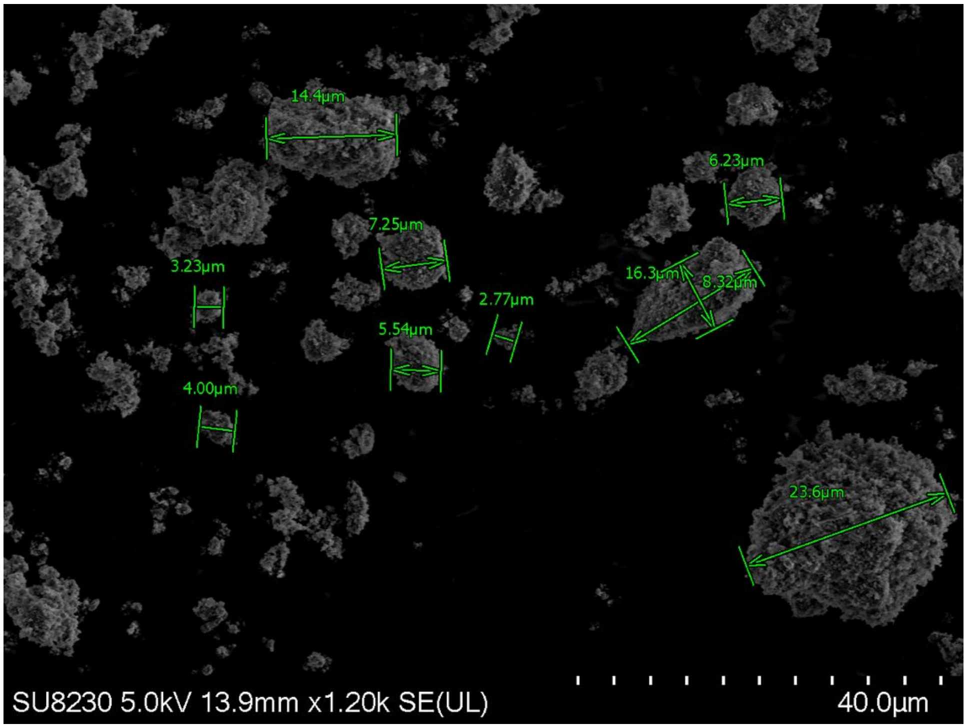

Figure S10. Measured particle sizes for 12h ball-milled HS-PEG using SEM. The approximate particle radius for this sample was estimated by halving the diameter of the largest particle in the image (~20 µm).

Adsorption Enthalpy

The enthalpy of adsorption for water onto HS-PEG and LiCl@HS-PEG samples was approximated through coupled differential scanning calorimetry and gravimetric analysis. Samples were analyzed in a Netzsch Jupiter F1 TGA/DSC equipped with a water vapor furnace and humidity generator. The raw data was processed with the Netzsch Proteus software.

All data collections were run under a constant gas flow of 200 ml/min, with the humidity controlled by the humidity generator. All samples were run through the same experiment profile: activation at 150 °C for 6 hours, controlled cooling to 27 °C, then a 6 hour adsorption step with a transition from 0 to 10 %RH 30 minutes into the adsorption step.

The enthalpy of adsorption was calculated through the integration of the calorimetric data over the course of the adsorption process to calculate the total energy of the adsorption process in Joules. The total quantity of the water adsorbed during the run was calculated from the gravimetric data and converted to moles.

Table S2: Water adsorption and enthalpy data for water adsorption in a humid stream of nitrogen, 10 %RH at 200 ml/min at 27 °C.

Sample	Loading (g/g)	Enthalpy (kJ/mol)
HS-PEG	0.028	85.7
HS-PEG (2xCTAB)	0.0158	84.7
LiCl@HS-PEG (2x CTAB) 30wt% MeOH	0.265	84.5
LiCl@HS-PEG (2x CTAB) 20wt% MeOH/H_2O	0.23	81.6
LiCl@HS-PEG (1x CTAB) 25wt% MeOH	0.349	80.6

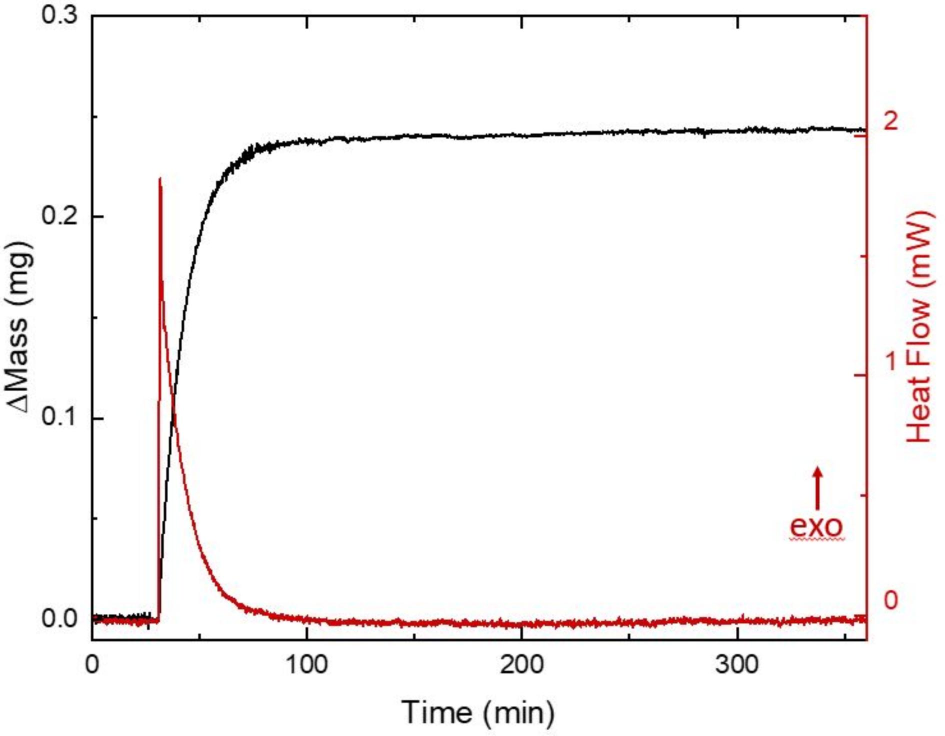

Figure S11: TGA/DSC data on the water adsorption of HS-PEG in a 200 ml/min flow of nitrogen at a constant 27 °C. The humidity of the gas flow was changed from 0 to 10 %RH 30 min into the adsorption step. Enthalpy of adsorption values were derived from the integration of the heat flow (red) and the quantity of water adsorbed (black). The enthalpy of adsorption was calculated to be 85.7 kJ/mol.

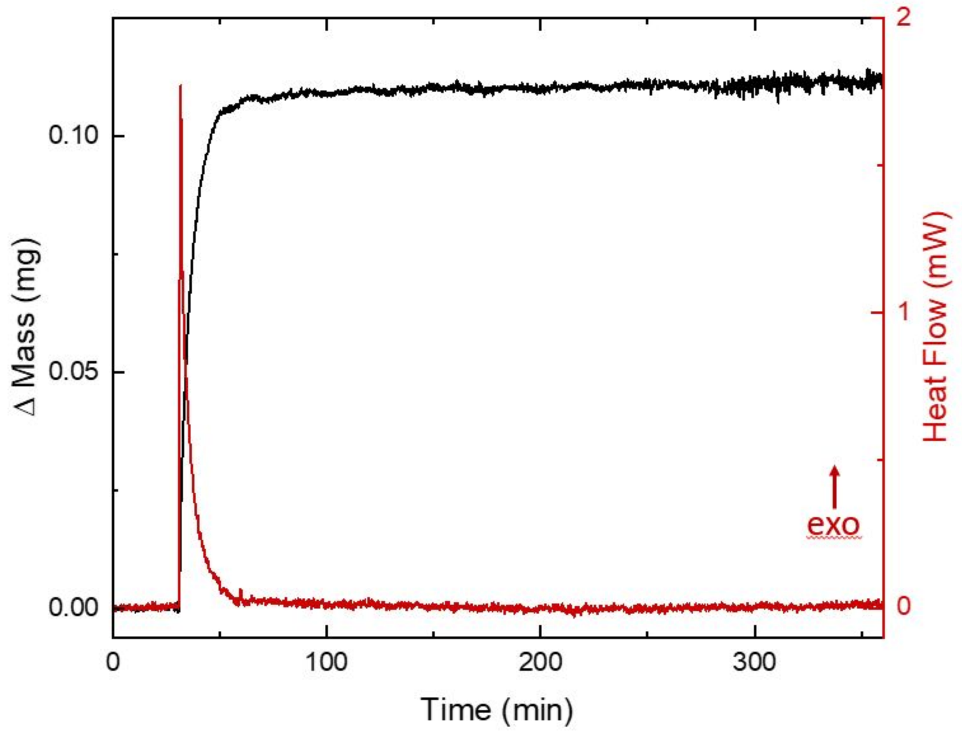

Figure S12: TGA/DSC data on the water adsorption of HS-PEG 2xCTAB in a 200 ml/min flow of nitrogen at a constant 27 °C. The humidity of the gas flow was changed from 0 to 10 %RH 30 min into the adsorption step. Enthalpy of adsorption values were derived from the integration of the heat flow (red) and the quantity of water adsorbed (black). The enthalpy of adsorption was calculated to be 84.7 kJ/mol.

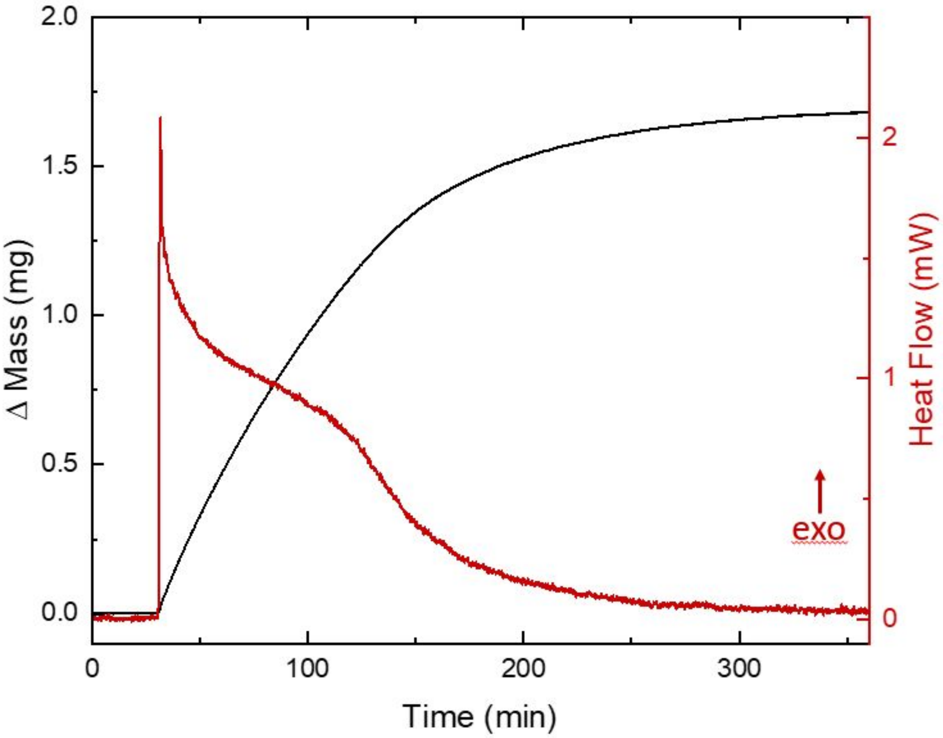

Figure S13: TGA/DSC data on the water adsorption of LiCl@HS-PEG 2xCTAB, 30 wt% in MeOH in a 200 ml/min flow of nitrogen at a constant 27 °C. The humidity of the gas flow was changed from 0 to 10 %RH 30 min into the adsorption step. Enthalpy of adsorption values were derived from the integration of the heat flow (red) and the quantity of water adsorbed (black). The enthalpy of adsorption was calculated to be 84.5 kJ/mol.

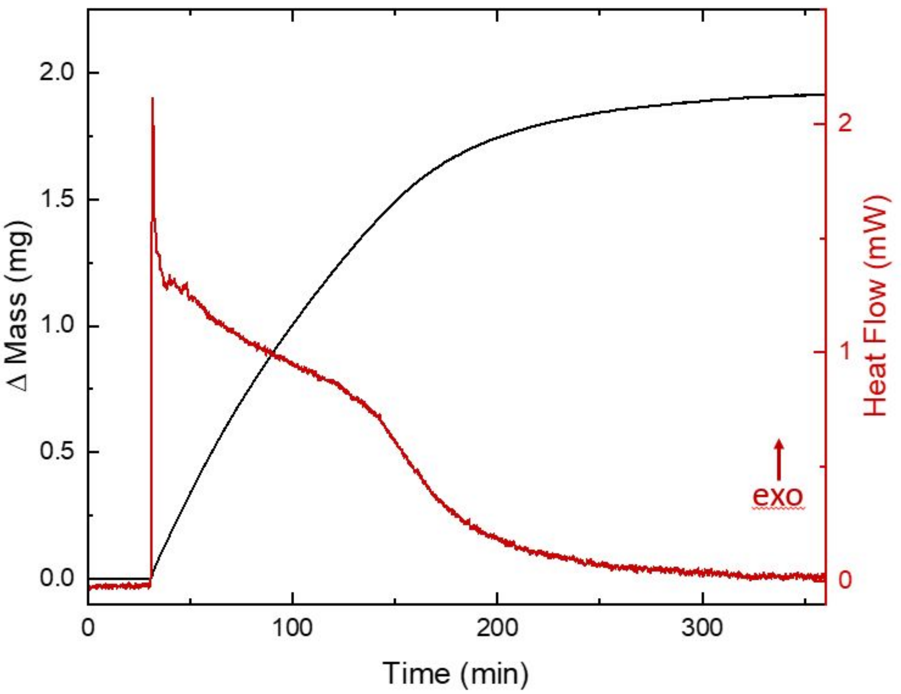

Figure S14: TGA/DSC data on the water adsorption of LiCl@HS-PEG 2xCTAB, 20 wt% in MeOH/H_2O in a 200 ml/min flow of nitrogen at a constant 27 °C. The humidity of the gas flow was changed from 0 to 10 %RH 30 min into the adsorption step. Enthalpy of adsorption values were derived from the integration of the heat flow (red) and the quantity of water adsorbed (black). The enthalpy of adsorption was calculated to be 81.6 kJ/mol.

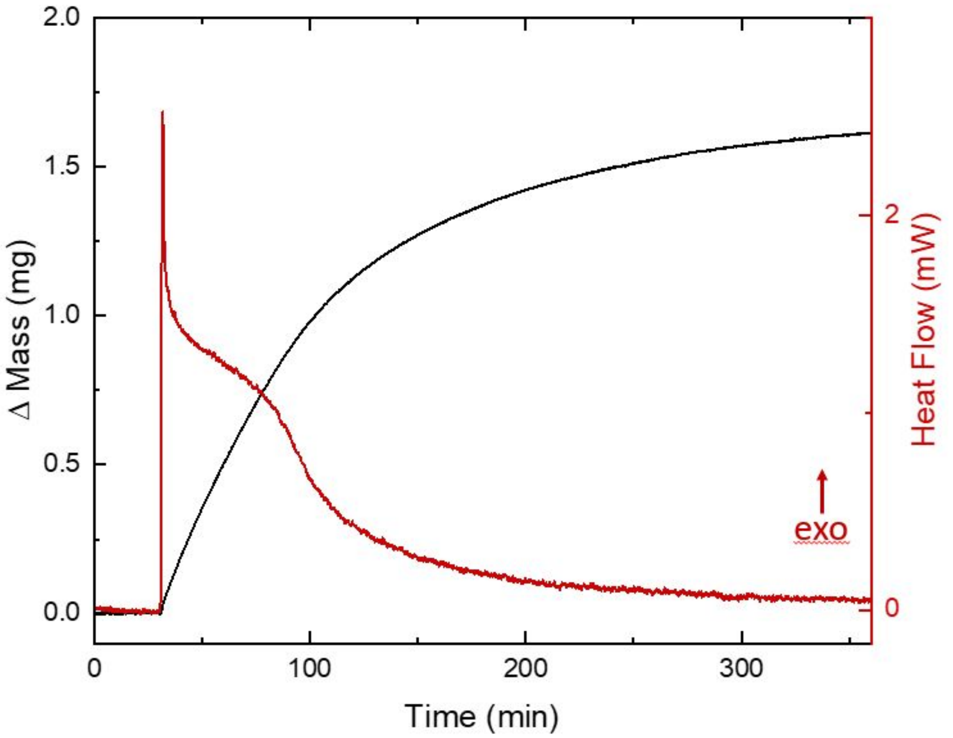

Figure S15: TGA/DSC data on the water adsorption of LiCl@HS-PEG (1x CTAB), 25 wt% in MeOH in a 200 ml/min flow of nitrogen at a constant 27 °C. The humidity of the gas flow was changed from 0 to 10 %RH 30 min into the adsorption step. Enthalpy of adsorption values were derived from the integration of the heat flow (red) and the quantity of water adsorbed (black). The enthalpy of adsorption was calculated to be 80.6 kJ/mol.
